# Risk Factors for Metastatic Castration-Resistant Prostate Cancer (CRPC) Predict Long-Term Treatment with Docetaxel

**DOI:** 10.1371/journal.pone.0048186

**Published:** 2012-10-30

**Authors:** Takashi Kawahara, Yasuhide Miyoshi, Zenkichi Sekiguchi, Futoshi Sano, Narihiko Hayashi, Jun-ichi Teranishi, Hiroshi Misaki, Kazumi Noguchi, Yoshinobu Kubota, Hiroji Uemura

**Affiliations:** 1 Department of Urology, Yokohama City University Graduate School of Medicine, Yokohama, Kanagawa, Japan; 2 Department of Urology, Yokohama City University Medical Center, Yokohama, Kanagawa, Japan; 3 Department of Urology, Yamato City Hospital, Yamato, Kanagawa, Japan; University of Colorado, United States of America

## Abstract

**Purpose:**

For patients with metastatic castration-resistant prostatic cancer (mCRPC), docetaxel plus prednisone leads to superior survival and a higher response rate compared with mitoxantrone plus prednisone. We analyzed the efficacy of long-term treatment with ≥10 cycles of docetaxel, and validated the risk group classification in predicting overall survival (OS) in Japanese patients with mCRPC.

**Patients and Methods:**

Fifty-two patients with mCRPC were administered 55 mg/m^2^ docetaxel and 8 mg dexamethasone, every 3 or 4 weeks, simultaneously with hormonal therapy and daily oral dexamethasone. They were divided into two groups, short-term (9 or fewer cycles) and long-term (10 or more cycles). Four risk factors including the presence of anemia, bone metastases, significant pain and visceral metastases were utilized for the risk group classification.

**Results:**

Fourteen patients (27%) had an elevation of PSA in spite of docetaxel treatment, while 23 patients (44%) had a decline in PSA level, including 9 patients (17%) whose PSA level declined by ≥50%. The median duration of OS after the initiation of this therapy was 11.2 months in the short-term group and 28.5 months in the long-term group. The good risk group showed a significant difference in OS compared with the intermediate and poor risk groups (P<0.001). The median number of cycles of treatment was 14, 4 and 3 for each risk group, respectively (p<0.01).

**Conclusions:**

The present study indicated that ≥10 cycles of this docetaxel therapy can significantly prolong survival in Japanese men with CRPC. This risk group classification for men with mCRPC at the initiation of this chemotherapy is useful.

## Introduction

Prostate cancer is the most common cancer in men, with approximately 220,000 cases and 29,000 deaths annually in the United States [1], [2]. In Japan, despite the lower incidence than in the United States, prostate cancer has steadily been increasing in recent years [2]. Huggins *et al.*, in 1941, first reported the efficacy of androgen deprivation therapy in advanced prostate cancer [3]. Although 80–90% of prostate cancers with metastatic lesions respond to initial androgen ablation therapy, most patients with prostate cancer ultimately develop progressive disease of hormone refractory cancer. Previous chemotherapy trials in CRPC have resulted in low response rates and minimal survival impact [4], and it is clear that better agents are needed to improve the outcome of chemotherapy in mCRPC patients. A regimen of docetaxel 75 mg/m^2^ once every 3 weeks with daily oral prednisone (PSL) based on the TAX327 trial has shown a significant survival advantage in patients with mCRPC as compared with mitoxantolone plus PSL [1]. Currently, docetaxel treatment has been established as standard chemotherapy for mCRPC, also in Japan. However, in the TAX327 study, there was no report of long-term use of docetaxel treatment for mCRPC. Furthermore, there is still much uncertainty regarding the timing of the initiation and discontinuation of this treatment.

Based on the fact that more than half of patients diagnosed with prostate cancer are aged more than 70 years, aggressive treatment such as docetaxel administration should be performed carefully in elderly patients with mCRPC. In fact, it is likely that many oncologists are reluctant to use the standard 3-week docetaxel regimen of 75 mg/m^2^ because of severe adverse events, such as neutropenia, which showed a significantly higher incidence compared to that with the weekly schedule [1], [5], [6], [7], [8]. Therefore, it is valuable to establish a tolerable regimen with a moderate dose of docetaxel for CRPC even for elderly patients, resulting in improved survival.

Many physicians have wondered as when to start docetaxel chemotherapy, because definitive prognostic factors at the initiation of docetaxel chemotherapy associated with disease progression and survival have not been identified. Recently, Armstrong *et al.* investigated pre-treatment factors that predicted PSA decline and overall survival in men treated with docetaxel chemotherapy by subgroup analysis of TAX327 [9]. Consequently, they reported that four independent risk factors (pain, visceral metastases, anemia and bone scan progression) predicted PSA decline and overall survival (OS). Furthermore, well-known prognostic factors, e.g. performance status, number of metastatic sites, baseline PSA and PSA doubling time, had independent prognostic importance in OS in men treated with docetaxel chemotherapy.

We have previously reported the efficacy and safety of docetaxel 55 mg/m^2^ treatment in Japanese mCRPC patients [10]. We then treated more patients with CRPC with further cycles of this treatment. In the present study, we compared the outcome and PSA change between the long-term (10 or more cycles) and short-term groups (9 or fewer cycles). Furthermore, we validated whether the above four prognostic factors could be applied to predict outcome in Japanese mCRPC patients treated with docetaxel chemotherapy.

## Patients and Methods

### Patient Selection

Fifty-two CRPC patients (median age: 65 years, range: 45–77 years) treated at Yokohama City University Hospital, Yokohama City University Medical Center and Yamato City Hospital were enrolled from 2003 to 2010. All patients were followed up for more than six months. This study was approved by the Institutional Review Board of Yokohama City University Hospital, Yokohama City University Medical Center and Yamato City Hospital. Written informed consent for this study was obtained from all patients. Subjects were included in the present study if they fulfilled the following eligibility criteria: (1) histologically confirmed prostate cancer; (2) prior exposure and failure of maximum androgen blockade therapy (MAB), (3) Eastern Cooperative Oncology Group (ECOG) performance status (PS) of 2 or better; (4) informed consent to this therapy.

In this study, the duration of tolerable cycles with docetaxel showed a bimodal distribution. Short-term treatment consisted of 7 or fewer cycles and long-term treatment consisted of 11 or more cycles. We therefore divided the subjects into two groups retrospectively; a short-term group who received up to 9 cycles of this therapy (31 patients) and a long-term group who received 10 or more cycles (21 patients).

### Drug Administration

Docetaxel 55 mg/m^2^ and dexamethasone 8 mg/body were given by intravenous infusion every 3–4 weeks. Subjects were simultaneously treated with hormonal therapy with an LHRH analogue and daily oral dexamethasone (0.5–1.0 mg/day). Before this treatment, 43 (83%) patients had received estramustine and all developed resistance to this drug. Docetaxel treatment at 55 mg/m^2^ was continued until the patient decided to stop, deterioration of general health due to disease progression or unacceptable toxicity occurred.

### Assessment

Patients underwent physical examination, complete blood count, liver function tests (aspartate transaminase [AST], alanine transaminase [ALT], alkaline phosphatase [ALP], bilirubin), and renal function tests (blood urea nitrogen [BUN], creatinine) as well as determination of PSA level before every start of docetaxel administration. Overall survival was also calculated, as the time from the first docetaxel administration to the time of death.

Risk factors were applied as determined by subgroup analysis of the TAX327 study [9]. Briefly, four pre-treatment risk factors that predicted PSA decline and OS in men with metastatic mCRPC included the presence of anemia, development of bone metastases, significant pain and the presence of visceral metastases. Among them, the criteria for anemia and development of bone metastases were modified to hemoglobin (Hb) less than 10 g/dL and alkaline phosphatase above the upper limit of normal at each hospital. We investigated the value of these four risk factors just before initial docetaxel therapy.

### Statistical Analysis

The Kaplan-Meier product limit estimator was used to estimate OS time. Survival duration was defined as the time between the first docetaxel administration and the time of death. All statistical computations were performed using the SPSS version 10.1 software package (SPSS Japan, Japan).

## Results

A total of 52 patients underwent this therapy. The pretreatment characteristics of the patients are listed in Table 1. The median age of all patients was 65 years (range: 49–77 years). Before docetaxel treatment, all patients had received MAB therapy, 43 patients (83%) had already received oral 280 mg estramustine and 0.5–1.0 mg dexamethasone, and 18 patients (35%) had received radiation therapy. None of the patients had received any cytotoxic drug or radiation therapy within at least one month before this treatment. Median serum PSA level was 161 ng/mL (range: 0.5–2305 ng/mL). All patients had metastatic lesions, which were in bone (n = 52), lung (n = 1), bladder (n = 2), and retroperitoneal lymph nodes (n = 9). The baseline characteristics of the two treatment groups, short-term and long-term treatment groups, were not significantly different in terms of age, serum PSA level, clinical stage, pathological grade and performance status (PS), as shown in Table 1.

**Table 1:** Baseline Characteristics of Patients.

Patient characteristics	All	Short term	Long term	P
No. of patients	52	31	21	
Median No. of cycles(range)	6 (2–40)	3 (2–7)	16 (15–40)	<0.001
Age				0.06
Median (yr)	65	68	64	
95% C.I. (yr)	67.0±1.8	68.4±2.2	65.0±3.1	
Pathological Grade				0.58
Gleason Score ≤7	14 (26.9%)	7 (22.6%)	7 (33.3%)	
Gleason Score = 8	20 (38.5%)	12 (38.7%)	8 (38.1%)	
Gleason Score ≥9	16 (30.8%)	10 (32.3%)	12 (28.6%)	
Unknown	2 (3.8%)	2 (6.4%)	0 (0%)	
Prior treatment				0.82
Maximal androgen blockade	52(100%)	31(100%)	21(100%)	
Prostatectomy	5 (10%)	2 (6%)	3 (14%)	
Radiotherapy	18 (35%)	10 (32%)	8 (38%)	
Estramustine	43 (83%)	23 (74%)	20 (95%)	
Serum PSA				0.17
Median (ng/ml)	151	237	81	
95% C.I. (ng/ml)	276.6±111.9	213.5±138.7	360.7±140.7	
Extent of disease				0.60
Bone	52 (100%)	31 (100%)	21 (100%)	
Lymph node	9 (17%)	7 (23%)	2 (10%)	
Bladder	2 (4%)	0 (0%)	2 (10%)	
Lung	1 (2%)	1 (3%)	0 (0%)	
Follow-up				0.16
Median (months)	27.5	32.0	23.0	
95% C.I. (months)	34.4±6.9	29.9±8.2	39.8±11.8	
Time from initial therapy to CRPC				0.11.
Median (months)	17.5	15.5	19	
95% C.I. (months)	23.3±5.8	18.8±7.1	28.1±9.4	

PSA; prostate specific antigen, CRPC; Castration resistant prostate cancer.

A decline in PSA was observed in 23 of 52 patients (44%), among whom nine patients (17%) had a 50% or greater decrease in PSA level from baseline and 14 patients (27%) had a less than 50% decrease. In the patients who received long-term treatment, seven patients (33%) had a 50% or greater decrease in PSA level from baseline, and 16 patients (76%) had a decrease from baseline including those patients with a 50% or greater decrease. On the other hand, in the short-term group, only two patients (6%) had a 50% or greater decrease in PSA level from baseline, and 5 patients (16%) had a 25% or greater decrease, including those patients with a 50% or greater decrease.

Although median overall survival after this therapy was 20.1 months in all patients, there was a significant difference between the short-term and long-term groups, 11.2 and 28.5 months, respectively (p<0.001) (data not shown). No significant difference was observed in the time from initial therapy to CRPC between the short-term and long-term groups, 15.5 and 19 months, respectively (Table 1). All patients in the short-term group had discontinued this therapy by 7 cycles.

**Figure 1:**
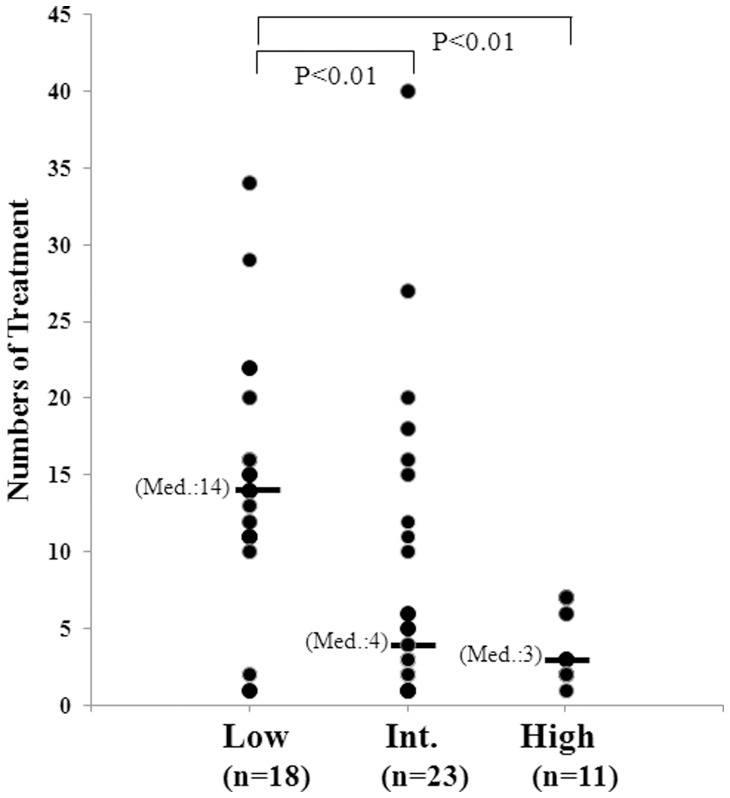
Docetaxel treatment number in each risk group with long-term treatment. The low risk group showed significant differences compared with the intermediate and high risk groups (P<0.01).

**Figure 2:**
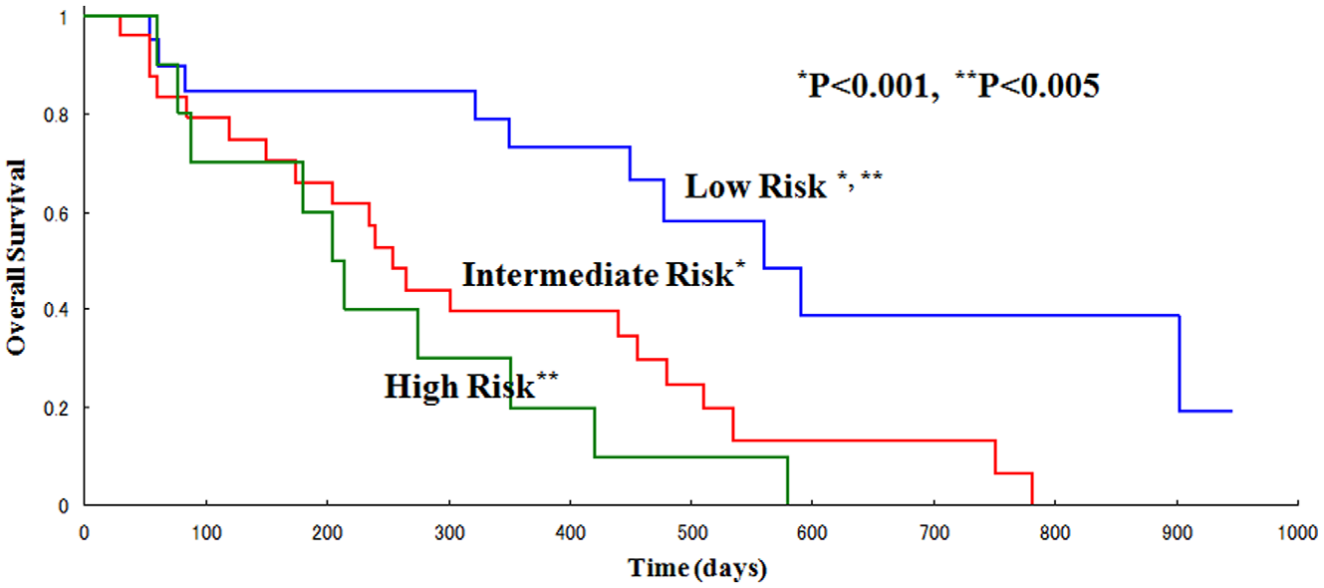
Overall survival in each risk group with long-term treatment. The low risk group showed significant differences compared with the intermediate and high risk groups (P<0.005 and P<0.001, respectively).

We stratified the patients into three cohorts with low risk (0 or 1 risk factor present), intermediate risk (2 risk factors present) and high risk (3–4 risk factors present). The risk factors in each risk group were shown in Table 2. We found a statistically significant difference in the number of cycles of treatment among risk groups (Fig. 1). Median treatment cycle number in each risk group was 14, 4 and 3 in the low, intermediate and high risk groups, respectively (p<0.01). Kaplan-Meier estimates of overall survival according to risk group classification are shown in Fig. 2. Median survival time was 560, 265 and 209 days in the low, intermediate and high risk groups, respectively (p<0.005).

**Table 2:** Distribution of risk factors classified by risk group.

	Low Risk	Intermediate Risk	High Risk
No. of Patients.	18	23	11
Pain	6 (33.3%)	18 (64.3%)	10 (90.9%)
visceral metastases	3 (16.7%)	4 (17.4%)	8 (72.7%)
Anemia	0 (0.0%)	4 (17.4%)	7 (63.6%)
Bone metastatis progression	2 (5.6%)	20 (87.0%)	11 (100.0%)

The most common toxicity was Grade 1 neutropenia, but no Grade 4 toxicity was observed. Hematological Grade 3 toxicity of neutropenia occurred in three patients (6%); two in the short-term group and one in the long-term group. The regimen was discontinued in one patient because of drug-induced interstitial pneumonia after the 4^th^ treatment in short-term group. Discontinuation of chemotherapy in most patients was because of deterioration of general health due to disease progressions, except in one case with interstitial pneumonia. None of the patients underwent dose reduction of docetaxel because of drug toxicity. Five patients with anemia were treated with blood transfusion (10%). Stomatitis was seen in one patient (2%), alopecia in seven (13%), sensory neuropathy in five (10%), and dyspnea in one (2%). There was a tendency for the long-term group to have more severe toxicity than the short-term group.

## Discussion

In the present study, docetaxel treatment at 55 mg/m^2^ with dexamethasone for mCRPC was performed from 2003. We previously reported nine mCRPC patients treated with docetaxel therapy, resulting in good efficacy [10]. We used dexamethasone instead of prednisone. We set the docetaxel therapy for CRPC in 2002 using docetaxel plus dexamethasone and confirmed the tolerance, effectiveness and safety. [10] Based on this previous evidence for the effectiveness for Japanese CRPC patients, we still used for dexamethasone instead of prednisone. We further accumulated 52 cases of CRPC patients who underwent docetaxel chemotherapy with the same regimen. It is noteworthy that 21 patients (41%) were able to receive 10 or more cycles with this protocol. Herein, we analyzed the efficacy and toxicity of long-term treatment with docetaxel in Japanese mCRPC patients. Although the overall survival in this study (20.1 months) was similar to that in the earlier report of updated survival analysis of the TAX327 study (docetaxel tri-weekly, median survival 19.2 months) [1], this study showed significantly longer overall survival (28.5 months) in the long-term group than in the short-term group (11.2 months). Pond et al reported that no survival benefit was seen with more than 10 cycles of tri-weekly docetaxel treatment [11]. In the present study, the limited sample size as well as the significantly better outcome of patients who received long-term docetaxel therapy might further limit the correct identification of indicators for performing this therapy in the long term.

From the results of this study, long-term chemotherapy improved the outcome of Japanese mCRPC patients. Therefore, it is important to determine if any patients could tolerate long-term docetaxel chemotherapy. Utilizing risk factors for survival that have been used to stratify men with mCRPC in clinical trials, we assessed whether the risk factors could be useful for predicting their outcome. As Armstrong *et al.* previously reported, four independent risk factors were identified: pain, visceral metastases, anemia and bone scan progression. Among them, because the hemoglobin level they proposed (<13 g/dL) seemed to be high for Japanese patients with long-term hormonal therapy, we modified it to <10 ng/dL. Like hemoglobin, alkaline phosphatase (ALP) is one of the classical prognostic survival factors in untreated patients with CRPC [12]. An increase in ALP level is thought to be a substitute for bone scan progression as a prognostic factor, and we therefore utilized it. Another bone marker including BAP and 1-CTP is clinically used. But, ALP is still a widely used marker in Japan and also in many countries. In some reports, the usefulness of ALP for evaluating bone metastasis is reported recently. [13] Therefore, we determined to use ALP as a surrogate marker of bone scan. A higher ALP level indeed was associated with a significant difference in OS in the present study (data not shown). As concerned about cut off point of ALP, both above the institutional upper normal limit and increasing value are adequate for clinical use. As expected, risk classification using these four factors indicated a significant difference in OS, which means that these risk factors should be criteria for making a decision on when to start docetaxel chemotherapy in mCRPC patients. We speculate that our risk factors could reflect tumor progression biologically.

Tannok *et al.* reported that the response rate for a >50% reduction of serum PSA level was 45% and the duration of survival was 18.9 months in the group with docetaxel 75 mg/m^2^ every 3 weeks, and 16.4 months in the mitoxantrone group in the TAX327 study [1]. Our response rate for a 50% reduction of serum PSA level (17%) was inferior to that in the TAX327 study because second hormonal therapy using oral dexamethasone had already been performed in all patients, and estramustine had been administered in most of the patients (83%). OS showed no significant difference between patients with previous use and no previous use of estramustine, with median OS of 18.7 months and 22.5 months, respectively (P = 0.50).

In the TAX327 study, tri-weekly docetaxel treatment showed higher OS (18.9 months) than weekly docetaxel treatment (17.4 months) [1]. In Japanese patients, tri-weekly docetaxel therapy also showed significantly better survival than weekly docetaxel therapy with median OS of 12.5 and 8.0 months, respectively [14]. Naito et al. reported the efficacy of docetaxel 70 mg/m^2^ every 3 weeks plus PSL 5 mg twice daily in Japanese CRPC patients. In that report, docetaxel treatment at 70 mg/m^2^ was effective not only for decreasing the PSA level, but also for decreasing the tumor mass using Response Evaluation Criteria in Solid Tumors (RECIST) analysis in prostate cancer. Regardless of the effectiveness, in 23 of 44 patients (52.2%) the dose of docetaxel had to be reduced because of side effects. As it was considered likely that the 70 mg/m^2^ regimen of docetaxel would be too much for Japanese CRPC patients, we decided on a lower dose of docetaxel of 55 mg/m^2^ [15]. As expected, the response rate of PSA in this study was indeed lower than those in earlier reports using 70 or 75 mg/m^2^ docetaxel; however, our low-dose regimen with 55 mg/m^2^ docetaxel was well tolerated in the long term [10].

OS rate in this study was similar to that in the TAX327 study, or even longer, especially in the long-term group (28.5 months). These results suggest that the response rate of PSA reduction would not necessarily be a reliable factor to predict the outcome in CRPC patients. In our study, after the first course, most patients received this treatment in an outpatient clinic. Grade 2 or 3 toxicity was seen more often in the long-term group than in the short-term group, but no Grade 4 toxicity was seen with our protocol. Therefore, we did not necessarily reduce the dose of docetaxel because of toxicity, except in one patient who developed interstitial pneumonia. These data suggest that long-term treatment with our protocol is well tolerated.

In the present study, docetaxel chemotherapy was continued for as long as possible because the enrolled patients had resistance to all treatments including initial and secondary hormone therapy (estramustine and dexamethasone) and irradiation. Recently, it has been argued that docetaxel chemotherapy should be utilized as soon as HRPC develops, based on the data of other solid tumors such as breast and lung cancer [16]. For example, in prostate cancer, the notion is that at initial hormone therapy, chemotherapy is thought to have a more profound cumulative effect because the burden of androgen-independent cells is low. However, as we have no useful options after the development of resistance to taxane-based chemotherapy, these chemotherapies at an early time point may fail to provide significant benefit, but only result in substantial toxicity. Therefore, it is possible that the long-term utility of docetaxel, such as shown in our study, will contribute to improving the survival of advanced CRPC patients.

Based on the present data of risk group classification, the introduction of chemotherapy is recommended to be at early stage in CRPC patients. In brief, the patients with asymptomatic condition and lesser disease burden had been tolerable for long-term chemotherapy. Regarding the discontinuance of chemotherapy, the adverse events and response to therapy are very important, while at least 3–4 cycles of docetaxel chemotherapy should be performed because the PSA flare-up phenomenon by initial chemotherapy is observed in some cases. The deterioration of general condition, serial abrupt increase of PSA and severe adverse events would compel the discontinuance of chemotherapy and changing alternative therapies. Currently in Japan, we have no choice but to continue this chemotherapy because new hormonal or chemotherapeutic agents have not been available. However, when several new agents such as abiraterone acetate, TAK700, MDV3100, cabazitaxel or alpharadin would be available, docetaxel chemotherapy will not be necessarily continued for a long term excluding the cases with constant very low PSA levels by chemotherapy.

In summary, although this was not a randomized retrospective study, administration of 55 mg/m^2^ docetaxel every 3 weeks and dexamethasone 8 mg/body was well tolerated for long-term treatment, resulting in an improvement in OS in Japanese CRPC patients. Risk factors that predicted OS in men with mCRPC with long-term docetaxel chemotherapy have been validated as a preliminary report.
